# Investigation of Creep Behavior of HDPE Pipe Butt Fusion Welded Joints Using a Stepped Isostress Method

**DOI:** 10.3390/polym16131803

**Published:** 2024-06-26

**Authors:** Chunmei Bai, Rong Lin, Huan Sheng Lai

**Affiliations:** 1 Architecture and Civil Engineering Institute, Guangdong University of Petrochemical Technology, Maoming 525000, China; 2 School of Chemical Engineering, Fuzhou University, Fuzhou 350116, China; 3 Sino-French Institute of Nuclear Engineering and Technology, Sun Yat-sen University, Zhuhai 519082, China

**Keywords:** HDPE, welded joint, stepped isostress method, butt fusion welding

## Abstract

The utilization of high-density polyethylene (HDPE) pipes is prevalent in water transportation due to their exceptional durability, resistance to corrosion, and ease of installation. Traditionally, butt fusion welding has been employed to connect HDPE pipes. In this study, scanning electron microscopy (SEM) was utilized to examine the microstructure of butt fusion welded joints of HDPE pipes, while the stepped isostress method (SSM) was employed to investigate their creep behavior at 100 °C in ambient air. SEM results revealed a significant presence of craze or lamellae in the base material, whereas minimal occurrences of craze or lamellae were observed in the melt zone. The results obtained from the SSM indicated that the creep life of butt fusion welded joints of HDPE pipes was not adversely affected by the welding bead, and their creep life was no less than that of the base material when ductile creep failure occurred.

## 1. Introduction

High-density polyethylene (HDPE) pipes are extensively utilized for water transportation in municipal engineering and are increasingly being adopted in nuclear power plants [1,2]. In comparison to conventional metal pipes, HDPE pipes offer numerous advantages including exceptional wear resistance, corrosion resistance, reliable connections, high toughness, scratch resistance, excellent crack-arresting performance, and prolonged service life [3]. Moreover, HDPE pipes are typically connected using butt fusion welding technology to form a robust welded joint that ensures the sealing and durability of the pipe systems. However, it is important to note that welded joints remain the weakest link in HDPE pipe systems, with a typical service life of 50 years or more [4,5]. Therefore, conducting an investigation into butt fusion welded joints of HDPE pipes holds significant importance in ensuring the safe and cost-effective operation of these pipelines.

The quality of butt fusion welded joints of HDPE pipes is influenced by several key factors, including heating temperature, welding pressure, endothermic time, and cooling time [6,7,8]. Additionally, inadequate homogenization during extrusion can also impact the quality of welded joints [9]. Various methods have been proposed for evaluating the mechanical properties of welded joints. Tensile and burst tests can be utilized to evaluate the short-term mechanical properties of welded joints [10,11,12,13]. Moreover, when it comes to assessing the effect of welding defects on the long-term performance of welded joints, the creep test proves more effective than hydrostatic testing [14,15,16]. However, it should be noted that creep tests at low stress levels typically require a substantial amount of time, often spanning several years.

HDPE is a kind of polymer. To minimize the duration of creep testing and accurately predict the creep life of polymers at low stress levels, several accelerated creep test methods have been proposed. The initial acceleration of the creep test is achieved through application of the time-temperature superposition principle (TTSP) [17]. Multiple specimens are subjected to testing at various experimental temperatures under a constant load in this method. Subsequently, a stepped isothermal method (SIM) was proposed [18]. This method involves subjecting a single specimen to testing under a constant load, while the temperature levels are incrementally increased until specimen failure occurs. Following this, the time-stress superposition principle (TSSP) was developed [19,20,21]. Multiple specimens are tested at different stress levels under a constant experimental temperature in this method. Finally, the stepped isostress method (SSM) is used as an accelerated technique [22,23]. A single specimen undergoes testing under a constant experimental temperature, while the stress levels are incrementally increased until specimen failure occurs. TTSP and SIM employ temperature as a means to accelerate the creep rate, thereby generating master curves at a stress level under a single or multiple reference temperatures. Conversely, TSSP and SSM utilize stress to accelerate the creep rate, resulting in master curves at a temperature level under a single or different reference stresses. In comparison to the SIM, the SSM proves more suitable for thicker specimens due to its elimination of the need for changing test temperatures and prevention of uneven temperature distribution caused by rapid specimen heating. The SSM has been successfully employed for investigating the creep properties of various polymers, including fibers [23], polyamide [24], and fiber-reinforced polymer composites [25]. This method holds potential for studying the creep behavior of butt fusion welded joints of HDPE pipes.

In this study, the microstructure of butt fusion welded joints of HDPE pipes was examined using scanning electron microscopy (SEM). Subsequently, the creep behavior of the base material and butt fusion welded joints of HDPE pipes was investigated using the SSM at a single experimental temperature level of 100 °C in ambient air. Finally, the creep behavior of the base material and butt fusion welded joints with and without welding bead was discussed.

## 2. Experiment

### 2.1. Material

This study employed HDPE PE100 water pipes, which were made by Fujian Sinyo Pipe Technology Co., Ltd., Fuqing, China. These pipes had an outer diameter of 200 mm and a standard dimension ratio (SDR) of 11, indicating that the ratio of the outer diameter to the wall thickness was 11. Hence, the wall thickness was 18.2 mm. The tensile behavior was investigated in our previous study at a temperature of 28 °C in ambient air, which resulted in a yield stress of 22.2 MPa, Poisson’s ratio of 0.4, and Young’s modulus of 1314 MPa [26]. The welding process used for joining these pipes followed the ISO 21307 standard, using a butt fusion welding machine (HDC 160-315, Huida Pipeline Technology Co., Ltd., Zhuji, China), and the main welding parameters are shown in Table 1 [27]. A detailed description of the welding process can also be found in our previous work [26]. In addition, the welded joints were meticulously polished to achieve a surface of high precision, followed by the application of pure gold spray for surface treatment. Subsequently, the microstructure of the welded joints was examined using scanning electron microscopy (SEM, TESCAN MIRA 3, Brno, Czech Republic), with an accelerating voltage of 10 kV and a beam intensity of 10 nA.

### 2.2. SSM Test

**Figure 1:**
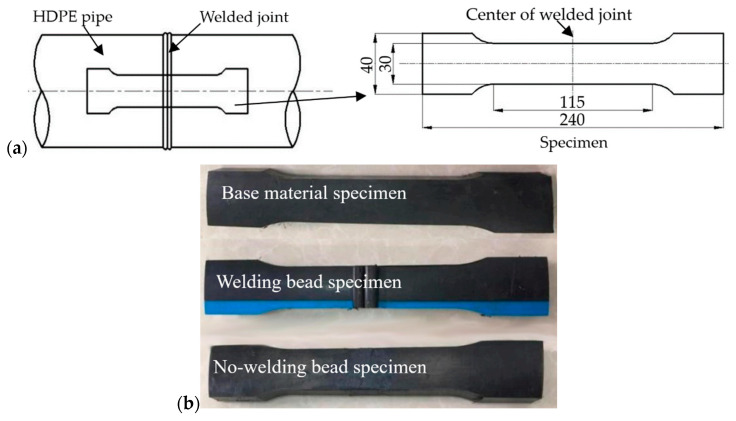
Specimens of SSM tests: (**a**) specimen preparation and dimensions (unit: mm), (**b**) three type specimens.

The test specimens were prepared in accordance with the BS EN 12814-3 standard [28], as shown in Figure 1a. The welding bead was positioned at the center of the specimen. Three distinct types of specimens were employed to investigate the long-term creep phenomenon of HDPE pipes, specifically focusing on the influence of welded joint and welding bead, as shown in Figure 1b. The first type encompassed base material specimens without any welded joints present. The second type consisted of specimens with both welded joints and welding beads, referred to as “welding bead specimens”. Lastly, the third type comprised specimens with welded joints but without the welding beads, termed “no-welding bead specimens”. Tests were carried out on a creep testing machine (DKRE50, Changchun Deli Measurement and Control Test System Co., Ltd., Changchun, China) with an environmental chamber. Temperature control within ±1 °C was achieved through utilization of the environmental chamber. An experimental temperature level of 100 °C was investigated in the ambient air. Prior to testing, the temperature was elevated to the experimental level and maintained for 30 min to ensure uniformity across all specimens.

The applied stress for all specimens in the initial step (step 1) was 3.5 MPa, as listed in Table 2. Therefore, the reference stress was set to 3.5 MPa. A total of five steps were employed, with an incremental stress increase of 0.5 MPa per step. The duration of each step from steps 1 to 4 was fixed at 5 h, while the test was terminated upon specimen failure during step 5. Minimization of the time interval between consecutive loading levels is crucial for mitigating the creep effect induced by loading steps. Therefore, a loading rate of 0.18 MPa/s was adopted in this study. The strain and testing time were automatically measured by the machine during the experiment. Two replicates were tested for each type of specimen.

## 3. Results and Discussion

### 3.1. Failure Specimens

**Figure 2:**
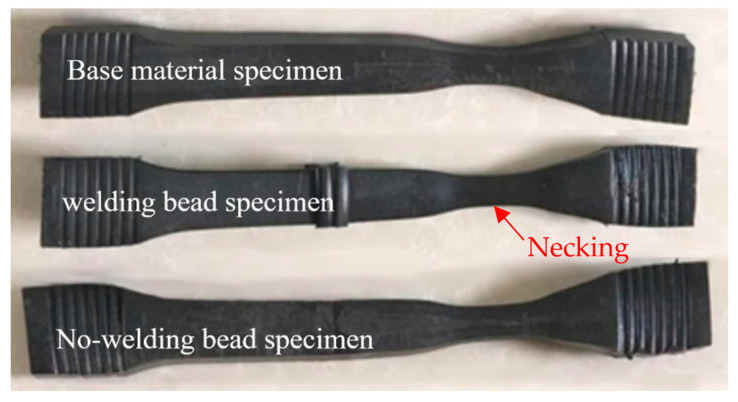
Typical failures of each type of specimen in the SSM tests at 100 °C in ambient air with a reference stress of 3.5 MPa.

**Figure 3:**
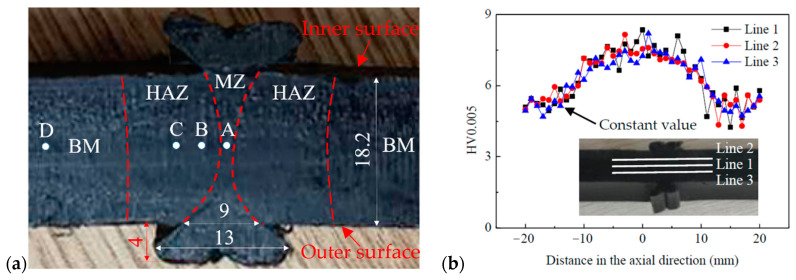
(**a**) Overview of butt fusion welded joints of HDPE pipe (unit: mm): D position (23 mm away from the weld center), C position (6 mm away from the weld center), B position (3 mm away from the weld center, and A position (on the weld center); (**b**) hardness distribution in the axial direction of a butt fusion welded joint of HDPE pipe [16].

The typical failure specimens are shown in Figure 2. All specimens exhibited necking rheological failure when necking occurred, indicating that all specimens experienced ductile failure. Furthermore, the welded joint demonstrated higher hardness compared to the base material, resulting in failure occurring away from the welded joint for both welding bead and no-welding bead specimens. However, it should be noted that even the base material specimen failed away from its center due to experimental setup constraints; theoretically, the base material specimen should fail at its center. In addition, the thickness of the no-welding specimen was equal to that of the pipe wall. The welding bead specimen consisted of two welding beads, located on the inner and outer surfaces, respectively. The dimensions of the welding bead are shown in Figure 3.

### 3.2. Microstructure Analysis

**Figure 4:**
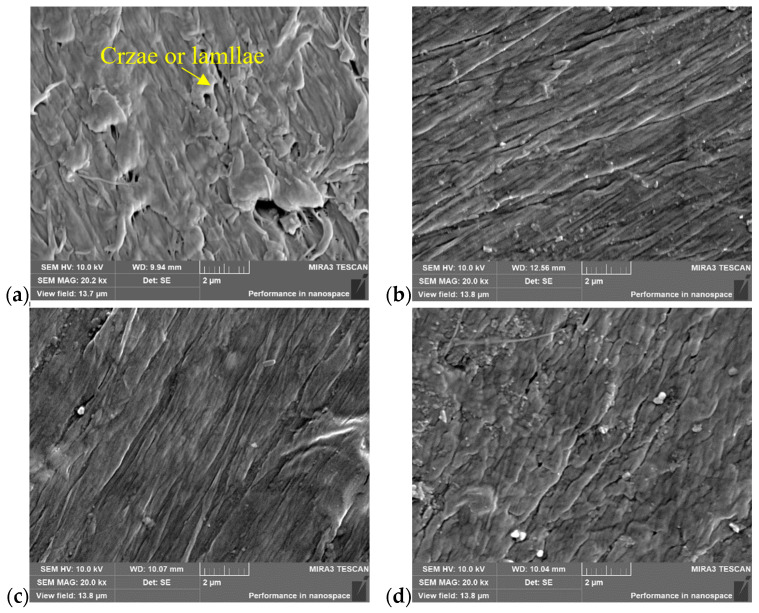
SEM images of butt fusion welded joints of HDPE pipe prior testing: (**a**) D position (23 mm away from the weld center), (**b**) C position (6 mm away from the weld center), (**c**) B position (3 mm away from the weld center, (**d**) A position (on the weld center).

The microstructure of the welded joint at locations A, B, and C, as well as that of the base material (BM) at location D, was investigated using SEM, as shown in Figure 3a. The locations A, B, C, and D were situated within the central region of the wall thickness. The borderlines of the melt zone (MZ) and the heat affect zone (HAZ) in Figure 3 were approximately determined based on the hardness distribution within the joint, as shown in Figure 3b [16]. The analysis results are shown in Figure 4 for a butt fusion welded joint that had not undergone any prior testing. Note that the welded joint underwent meticulous polishing prior to SEM analysis. Notably, numerous crazes or lamellae were observed at location D in BM, as shown in Figure 4a. However, these crazes exhibited a significant reduction within the HAZ, as shown in Figure 4b,c. Conversely, almost no craze or lamellae were found; instead, a substantial presence of grain-like structures was evident within the MZ, as shown in Figure 4d. Consequently, the distinct microstructure at different locations of the welded joint resulted in variations in the mechanical properties across these regions, such as hardness and elastic modulus. Previous studies have reported that the MZ exhibits superior hardness levels along with higher degrees of crystallinity and Young’s modulus [29].

**Figure 5:**
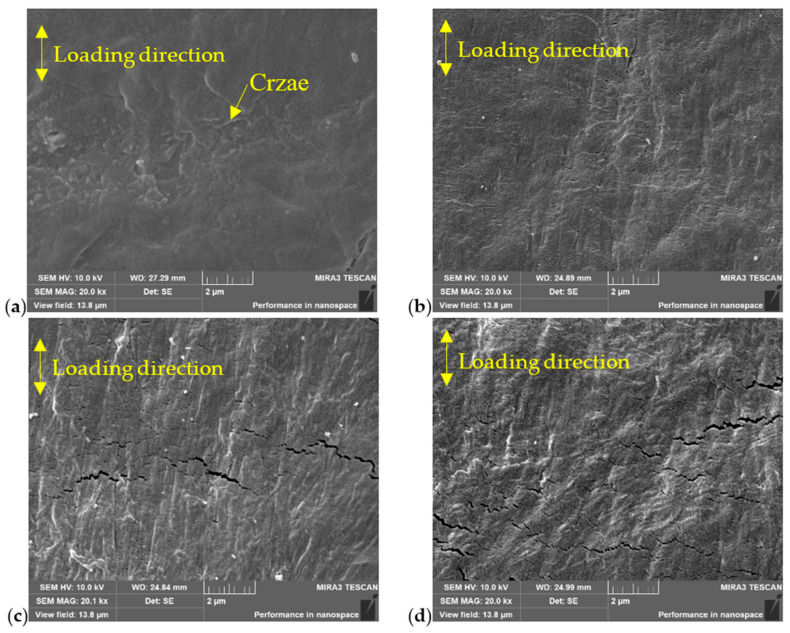
Typical SEM images of butt fusion welded joint of HDPE pipes for a failed welding bead specimen: (**a**) D position (23 mm away from the weld center), (**b**) C position (6 mm away from the weld center), (**c**) B position (3 mm away from the weld center, (**d**) A position (on the weld center).

**Figure 6:**
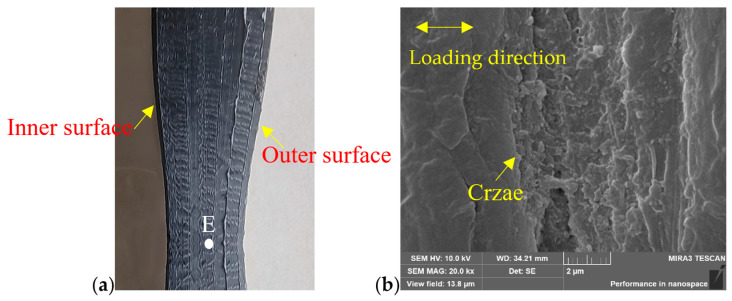
Typical SEM image on the out surface of the necking region of a failed welding bead specimen: (**a**) necking region, (**b**) SEM image of the E position.

Since none of the specimens failed at the butt fusion welded joint, as shown in Figure 2, SEM was used to investigate the failed specimen on the out surface of the welded joint at different locations, which were the same as shown in Figure 3a. The typical results of SEM images on the out surface of a butt fusion welded joint at different locations are shown in Figure 5 for a failed welding bead specimen. As shown in Figure 5a, fracture crazes were observed in BM, while Figure 5b reveals numerous micro-cracks in the HAZ. Moving closer to the welding center, as shown in Figure 5c,d, both the number and length of cracks increased significantly. The no-welding bead specimen exhibited a similar microstructure on the outer surface of the welded joint at various locations. SEM was also used to investigate the location of point E on the out surface of the necking region of a failed welding bead specimen, as shown in Figure 6a. A significant amount of fracture crazes were observed in the necking region, as shown in Figure 6b. Note that the welding bead and no-welding bead specimens failed away from the welding joint and experienced necking in the base material, as shown in Figure 2. Hence, the SEM images on the outer surface of the necking region were found to be identical for all three types of specimens.

### 3.3. Strain–Time Curves

**Figure 7:**
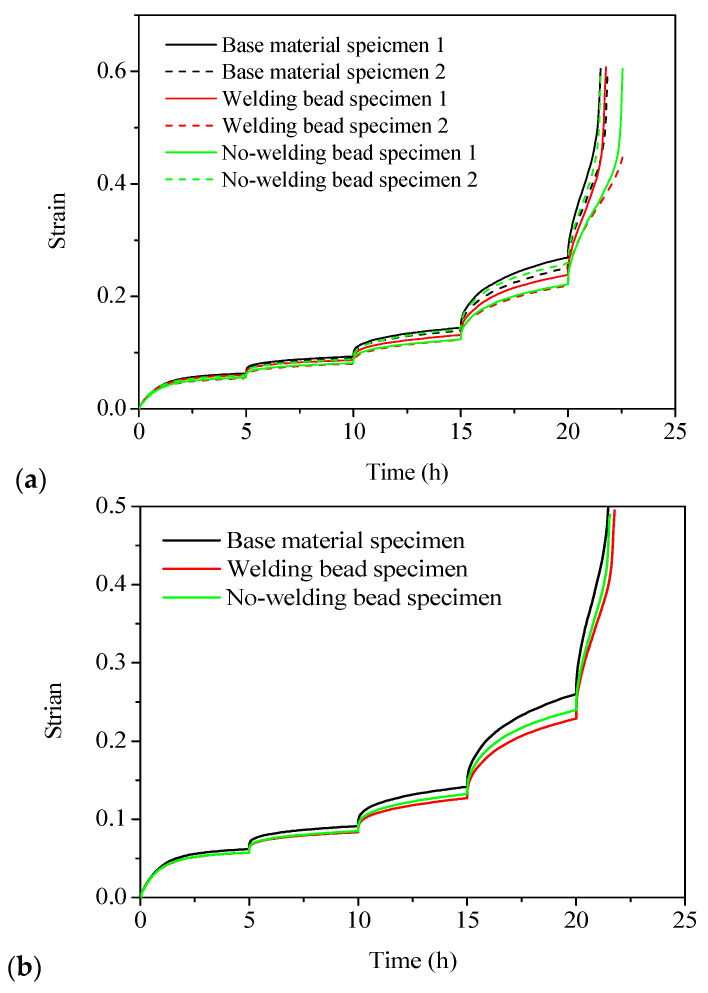
SSM test results of strain versus time curves at 100 °C in ambient air with a reference stress of 3.5 MPa: (**a**) test results for all specimens, (**b**) average curve of each type of specimen.

The SSM test results for all specimens are shown in the strain versus time curves in Figure 7a. Two replicates were tested, and excellent reproducibility was observed for each type of specimen, as shown in Figure 7a. In addition, the difference between the two curves of the base material specimens was minimal, but there was a slight disparity observed for the welding bead and no-welding bead specimens. This discrepancy can be primarily attributed to the slightly non-uniform quality of the welded joint. Therefore, an average curve was derived from each type of specimen, as shown in Figure 7b, to generate the master curve using the SSM in the subsequent section.

**Figure 8:**
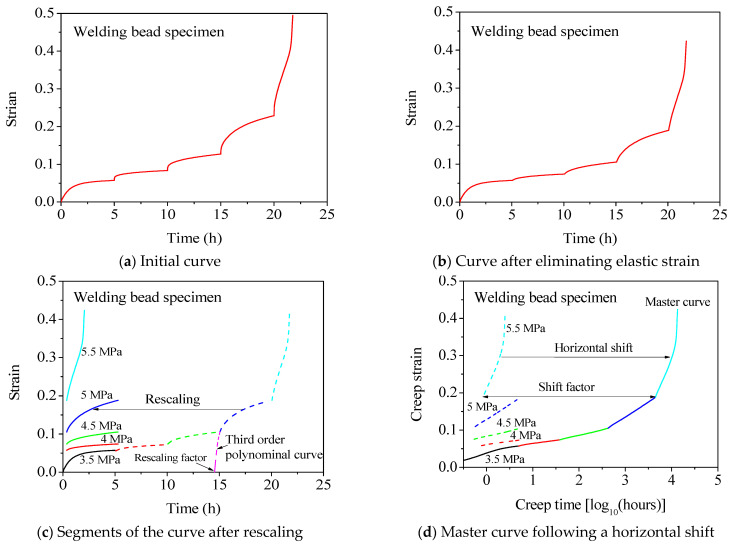
Data processing of the welding bead specimen of HDPE pipe at 100 °C in ambient air with a reference stress of 3.5 MPa, using the methodology of SSM.

The data processing procedure was presented as follows to demonstrate the application of the methodology of SSM for obtaining the master curve of the welding bead specimen, as shown in Figure 8. Firstly, the initial curve shown in Figure 8a was vertically adjusted to eliminate the elastic strain, thereby obtaining a pure creep curve, as shown in Figure 8b. Subsequently, a third-order polynomial function was employed to rescale the curve, as shown in Figure 8c. The third-order polynomial function was defined as follows:

(1) $$\varepsilon =at+bt^{2}+ct^{3}+d$$

where ε is the strain, *t* is the time, and *a*, *b*, *c*, and *d* are the fitted factors. The values of *a*, *b*, *c*, and *d* are determined by utilizing Equation (1) to fit the time–strain curve within the time period ranging from 1 h before to 0.5 h after the stress step. The value of the rescaling factor *r* corresponds to one of the solutions of the following equation:

(2) $$at+bt^{2}+ct^{3}+d=0$$

There were three solutions in Equation (2) theoretically, but only one solution was slightly less than the time at the corresponding incremental stress step, and this solution was the value of *r*, as shown in Figure 8c. When the time value of the curve was subtracted from the corresponding value of *r* at each stress level, the curve was horizontally shifted from right to left along the time axis. By performing this rescaling process, distinct segments of the curve corresponding to different stress levels were obtained, as shown in Figure 8c.

The segments were plotted on a new coordinate system featuring a logarithmic time axis, as shown in Figure 8d. Finally, these segments were horizontally shifted from left to right along the logarithmic time axis, and then connected end to end in order to derive the ultimate master curve, as shown in Figure 8d. The value of shift factor $\alpha_{\sigma }$ corresponded to the magnitude of movement along the logarithmic time axis for each segment, as shown in Figure 8d. All values of *r* and $\alpha_{\sigma }$ are listed in Table 3. Note that rescaling and horizontal shifting can be executed visually, but a numerical procedure can also be used at each stress increment to obtain a smooth master curve. Therefore, a third-order polynomial function was used to determine the value of *r*, and the master curve was aligned seamlessly with these segments end-to-end due to the high degree of smoothness exhibited by the resulting master curve. More detailed instructions on utilizing the SSM can be found in the reference [22].

**Figure 9:**
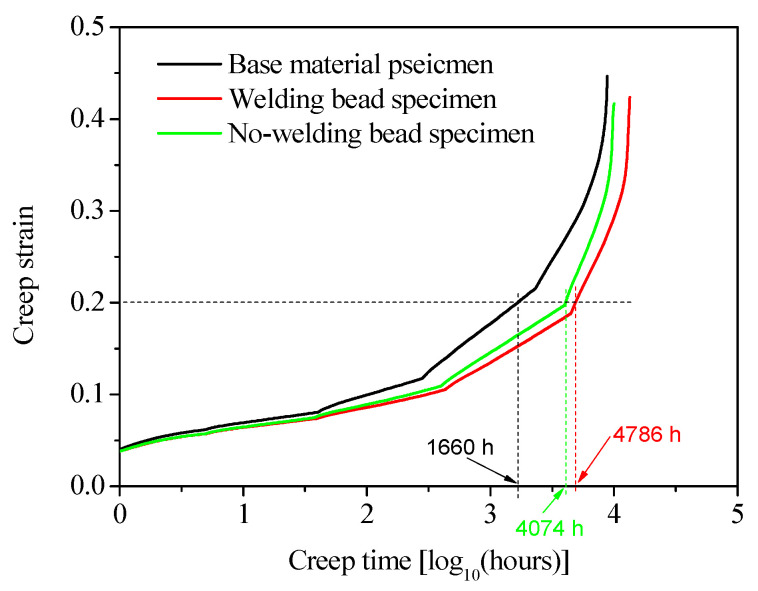
Master curves of HDPE pipes at 100 °C in ambient air with a reference stress of 3.5 MPa.

The data of the base material and no-welding bead specimens were processed using the same method as that for the welding bead specimen. Their values of *r* and $\alpha_{\sigma }$ are listed in Table 3, while the final master curves are shown in Figure 9 for all three types of specimens. As shown in Figure 9, the curve representing the base material specimen exhibited a higher magnitude compared to that of the welding bead specimen due to its inherent hardness, as shown in Figure 2. The curve of the no-welding bead specimen was positioned between the curves of the base material and welding bead specimens. If it was assumed that creep failure occurred when the creep strain reached a maximum value of 0.2 for all specimens, the creep life can be determined from Figure 9; the creep life of the base material specimen was 1660 h, while those of the welding bead and no-welding bead specimens were 4786 h and 4074 h, respectively. Therefore, it can be concluded that the presence of a welding bead does not adversely affect the creep life of welded joints, which is comparable to that of the base material. In fact, the creep life of welded joints was evidently longer than that observed for the base material when creep ductile failure occurred. However, the validity of this conclusion is limited to the occurrence of creep ductile failure. Investigation of the creep life of welded joints and the influence of welding beads on creep life in cases of brittle creep failure necessitates further research.

## 4. Conclusions

The microstructure of base material and butt fusion welded joints of HDPE pipes was investigated using SEM, while their creep behavior was analyzed using SSM tests at a temperature of 100 °C in ambient air. The following conclusions were drawn:

(1) SEM analysis revealed that the base material of HDPE pipes exhibited a significant presence of craze or lamellae, which were less prevalent in the heat-affected zone and even less present in the melt zone.
(2) SSM test results demonstrated that HDPE pipes had a creep life exceeding 1000 h when subjected to a stress of 3.5 MPa at a temperature of 100 °C in ambient air.
(3) SSM test results indicated that welding beads did not adversely affect the creep life of butt fusion welded joints of HDPE pipes; in fact, their creep life was no less than that observed for the base material when creep ductile failure occurred.

## Figures and Tables

**Table 1:** Butt fusion welding parameters for HDPE pipes [27].

Heating Temperature (°C)	Endothermic Time (s)	Cooling Time (s)	Flanging Pressure (MPa)	Welding Pressure (MPa)
210	140	960	0.15	0.15

**Table 2:** Experimental parameters of SSM tests at 100 °C in ambient air.

Step	1	2	3	4	5
Stress (MPa)	3.5	4	4.5	5	5.5
Elapsed time (h)	5	5	5	5	Until fail

**Table 3:** Rescaling and shift factors of different specimens at 100 °C in ambient air with a reference stress of 3.5 MPa.

Step	Rescaling Factor *r* (h)			$\mathrm{Shift}\;\mathrm{Factor}\;\log(\alpha_{\sigma }$)		
	Base Material Specimen	Wedling Bead Specimen	No-Welding Bead Specimen	Base Material Specimen	Wedling Bead Specimen	No-Welding Bead Specimen
1	/	/	/	/	/	/
2	4.37	4.34	4.31	0.85	0.83	0.82
3	9.25	9.58	9.56	1.69	1.90	1.87
4	14.39	14.55	14.52	2.62	2.92	2.86
5	19.57	19.22	19.09	3.66	3.72	3.61

## Data Availability

The original contributions presented in the study are included in the article, further inquiries can be directed to the corresponding author.
